# Calculating expected years of life lost for assessing local ethnic disparities in causes of premature death

**DOI:** 10.1186/1471-2458-8-116

**Published:** 2008-04-10

**Authors:** Tomás J Aragón, Daphne Y Lichtensztajn, Brian S Katcher, Randy Reiter, Mitchell H Katz

**Affiliations:** 1 Division of Epidemiology, School of Public Health, University of California, Berkeley, California, USA; 2 Community Health Epidemiology, San Francisco Department of Public Health, San Francisco, California, USA; 3 Department of Epidemiology and Biostatistics, University of California, San Francisco, California, USA

## Abstract

**Background:**

A core function of local health departments is to conduct health assessments. The analysis of death certificates provides information on diseases, conditions, and injuries that are likely to cause death – an important outcome indicator of population health. The expected years of life lost (*YLL*) measure is a valid, stand-alone measure for identifying and ranking the underlying causes of premature death. The purpose of this study was to rank the leading causes of premature death among San Francisco residents, and to share detailed methods so that these analyses can be used in other local health jurisdictions.

**Methods:**

Using death registry data and population estimates for San Francisco deaths in 2003–2004, we calculated the number of deaths, *YLL*, and age-standardized *YLL *rates (*ASYR*s). The results were stratified by sex, ethnicity, and underlying cause of death. The *YLL *values were used to rank the leading causes of premature death for men and women, and by ethnicity.

**Results:**

In the years 2003–2004, 6312 men died (73,627 years of life lost), and 5726 women died (51,194 years of life lost). The *ASYR *for men was 65% higher compared to the *ASYR *for women (8971.1 vs. 5438.6 per 100,000 persons per year). The leading causes of premature deaths are those with the largest average *YLL*s and are largely preventable. Among men, these were HIV/AIDS, suicide, drug overdose, homicide, and alcohol use disorder; and among women, these were lung cancer, breast cancer, hypertensive heart disease, colon cancer, and diabetes mellitus. A large health disparity exists between African Americans and other ethnic groups: African American age-adjusted overall and cause-specific *YLL *rates were higher, especially for homicide among men. Except for homicide among Latino men, Latinos and Asians have comparable or lower *YLL *rates among the leading causes of death compared to whites.

**Conclusion:**

Local death registry data can be used to measure, rank, and monitor the leading causes of premature death, and to measure and monitor ethnic health disparities.

## Background

A core function of local health departments is to conduct public health surveillance, including population health assessments [1,2]. Public health surveillance is the ongoing, systematic collection, analysis, interpretation, and dissemination of data regarding a health-related event for use in public health action to reduce morbidity and mortality and to improve health [3]. For a local health jurisdiction, primary data collection, such as representative population-based surveys, can be expensive and unsustainable. Therefore, local health departments must analyze existing health data, preferably those that are population-based, comprehensive, readily available, and locally relevant.

Death records are an important data source for assessing population health and health disparities because they cover the whole population and include information on key characteristics of decedents, including age, sex, ethnicity, place of residence and of death, and underlying and contributing causes of death. However, cause-specific mortality is typically reported using traditional epidemiologic measures, especially counts and rates (including age-adjusted rates), that are heavily influenced by deaths among older residents. For most causes, these measures are not very sensitive to deaths occurring at younger ages, which are more likely to be premature, preventable deaths.

To identify and prioritize causes of premature death, the *standard expected years of life lost *(*YLL*) measure, as developed by the Global Burden of Disease Study [4], provides a valuable analytic tool which can be applied to local geographic areas. The *YLL *is based on comparing the age of death to an external standard life expectancy curve, and can incorporate time discounting and age weighting. The *YLL*, combined with the years lived with disability (*YLD*) measure, make up the disability-adjusted life year (*DALY*). Unfortunately, directly measuring *YLD*s (and therefore *DALY*s) is cost prohibitive and not practical for most local health jurisdictions. In contrast, *YLL*s are measurable for a comprehensive set of conditions. *YLL*s, as opposed to more traditional mortality measures (counts, rates, etc.), highlight premature deaths. Where population estimates are available, age-adjusted *YLL *rates allow comparisons across groups or over time [5]. These deaths are particularly important from a public health and public policy perspective because they represent preventable loss of life.

Although the *YLL *is a valid, stand-alone measure for identifying and ranking the causes of premature death for a region [6-8], this measure has not been widely adopted for local area mortality analyses. There are several reasons for this. First, detailed methods for calculating *YLL *are not available in standard epidemiology textbooks or scientific journal articles. In contrast, the years of potential life lost (*YPLL*) is commonly used: it is easily calculated by subtracting the age of death from a chosen cut-off (e.g., 65, 75, or 85 years) [9,10]; however, the *YPLL *does not measure deaths after the cut-off age, and it does not incorporate time-based discounting used in cost-effectiveness analysis. The *YLL *measures every death and can incorporate discounting. Second, with few exceptions [5], sufficient local area *YLL *analyses have not been published to demonstrate their value in assessing population health. And third, readily available software solutions to make analyses more efficient have not been developed.

The purpose of this paper is to provide detailed methods for calculating *YLL *for a local geographic area (San Francisco, California, United States), and to demonstrate its value as a population health measure to impact local public health priorities. We illustrate how to use *YLL *to rank causes of death, and how to use average *YLL*s to identify the leading causes of premature death for major ethnic groups. Analysis and interpretation of death registry data using *YLL*s provide objective evidence for public health policymakers, partners, and stakeholders to inform and guide the setting of local public health priorities. This is especially important because of the geographic and demographic variation in health outcomes, major risk factors, and health disparities [5,11].

## Methods

### Overview

Summarized in Table 1 are the notation and definitions used in this article. For the years 2003–2004, registered deaths for San Francisco were obtained from the State of California, Department of Health Services, Center for Health Statistics [12]. The data file contained the underlying causes of death of San Francisco residents (whether or not they died in San Francisco), and the underlying causes of death of non-residents that died in San Francisco. For this study, only San Francisco resident underlying causes of death were used. Population estimates were obtained from the State of California, Department of Finance, Demographic Research Unit [13]. Standard life expectancies for men and women are from the Coale-Demeny Model Life Tables West: Levels 25 for men, and Level 26 for women (Table 2). For calculating age-standardized rates we used the Year 2000 United States standard million population (Table 3).

**Table 1:** Summary of notation and definitions

Notation	Definition
Introduced in Equation 1	
*x*	Age
*n*	Age interval length
*e*_*x*_	Life expectancy at age *x*
$e_{x}^{s}$	Standard life expectancy at age *x*
${\;}_{n}e_{x}^{s}$	Standard life expectancy for age interval *x *to *x *+ *n*
_*n*_*a*_*x*_	Average age of death for age interval *x *to *x *+ *n*
Introduced in Equations 2 to 5	
_*n*_*Y*_*x*_	Expected years of life lost for age interval *x *to *x *+ *n*
_*n*_*D*_*x*_	Number of deaths in age interval *x *to *x *+ *n*
*r*	Discount rate (usually set to 0.03)
K	Age-weighting modulation constant (*K *= 0, no weighting; *K *= 1, weighting)
*β*	Age-weighting constant (set to *β *= 0.04)
*C*	Adjustment constant for age-weights (set to 0.1658)
*YLL*	Expected years of life lost (*Y LL *= ∑_*n*_*Y*_*x*_)
Equation 6	
*ASYR*	Age-standardized *Y LL *rate (∑${\;}_{n}y_{x}^{s}$)
${\;}_{n}y_{x}^{s}$	Weighted expected years of life rate for age interval *x *to *x *+ *n*
_*n*_*y*_*x*_	Expected years of life lost rate for age interval *x *to *x *+ *n*
_*n*_*N*_*x*_	Population (person-year) estimate for age interval *x *to *x *+ *n*
_*n*_*w*_*x*_	Year 2000 United States standard population as weights (∑_*n*_*w*_*x *_= 1)
_*n*_*W*_*x*_	Year 2000 United States standard population (∑_*n*_*W*_*x *_= 1,000,000)

**Table 2:** Standard life expectancies based on Model Life Table West, Level 25 and 26

Age (*x*)	Level 25: Male life expectancy ($e_{x}^{s}$)	Level 26: Female life expectancy ($e_{x}^{s}$)
0	80.000	82.500
1	79.358	81.840
5	75.383	77.950
10	70.400	72.990
15	65.414	68.020
20	60.438	63.080
25	55.471	58.170
30	50.512	53.270
35	45.565	48.380
40	40.641	43.530
45	35.766	38.720
50	30.990	33.990
55	26.322	29.370
60	21.810	24.830
65	17.499	20.440
70	13.577	16.200
75	10.166	12.280
80	7.447	8.900
85	5.238	6.220
90	3.544	4.250
95	2.311	2.890

**Table 3:** Year 2000 United States standard million population

Age interval	_*n*_*W*_*x*_	_*n*_*w*_*x*_
< 1	13,818	0.013818
1–4	55,317	0.055317
5–14	145,565	0.145565
15–24	138,646	0.138646
25–34	135,573	0.135573
35–44	162,613	0.162613
45–54	134,834	0.134834
55–64	87,247	0.087247
65–74	66,037	0.066037
75–84	44,842	0.044842
85+	15,508	0.015508

The deaths and population estimates were aggregated into 19 age intervals (columns 1 and 2 of Table 4). For age-standardizations, 11 age intervals were used (Tables 3 and 5). The 19-level age intervals were used for calculating expected years of life lost (*YLL*) for men and women, (Table 4), stratified by sex and ethnicity (Table 6), stratified by cause of death and sex (Table 7), and stratified by cause of death, sex, and ethnicity (Additional file 1: Tables A-1 to A-4).

**Table 4:** Calculating expected years of life lost for San Francisco men and women, 2003–2004

		Male				Female			
			
Age interval	*n*	_*n*_*D*_*x*_	_*n*_*a*_*x*_	${\;}_{n}e_{x}^{s}$	_*n*_*Y*_*x*_	_*n*_*D*_*x*_	_*n*_*a*_*x*_	${\;}_{n}e_{x}^{s}$	_*n*_*Y*_*x*_
< 1	1	28	0.1	80.0	848.6	30	0.1	82.4	915.6
1–4	4	10	2.4	78.0	301.2	3	3.3	79.6	90.8
5–9	5	3	6.4	74.0	89.1	5	9.3	73.7	148.4
10–14	5	4	13.3	67.1	115.5	2	12.9	70.1	58.5
15–19	5	27	17.9	62.5	762.1	6	17.7	65.3	171.8
20–24	5	56	22.6	57.9	1,538.0	16	22.6	60.5	446.5
25–29	5	72	27.4	53.1	1,912.6	27	27.1	56.1	732.8
30–34	5	111	33.0	47.5	2,810.4	32	32.6	50.7	833.6
35–39	5	156	37.7	42.9	3,764.2	49	37.2	46.2	1,224.9
40–44	5	249	42.6	38.1	5,655.8	79	42.8	40.8	1,859.5
45–49	5	325	47.6	33.3	6,844.8	141	47.7	36.2	3,113.3
50–54	5	434	52.5	28.6	8,340.5	188	52.7	31.5	3,833.6
55–59	5	454	57.5	24.1	7,782.5	217	57.5	27.1	4,022.4
60–64	5	403	62.4	19.7	5,999.3	214	62.3	22.8	3,534.4
65–69	5	465	67.7	15.4	5,738.5	263	67.5	18.3	3,709.9
70–74	5	547	72.6	11.8	5,432.1	406	72.7	14.0	4,653.9
75–79	5	787	77.6	8.7	6,054.1	697	77.7	10.5	6,258.3
80–84	5	887	82.5	6.3	5,117.3	948	82.6	7.5	6,385.5
85+	10	1,294	90.3	3.7	4,520.3	2,403	91.5	4.1	9,200.3

Total	95	6,312 (Deaths)			73,626.8 (*YLL*)	5,726 (Deaths)			51,194.2 (*YLL*)

**Table 5:** Calculating direct age-standardized expected years of life lost rate for San Francisco men and women, 2003–2004

Age interval	Male				Female			
		
	_*n*_*N*_*x*_	_*n*_*Y*_*x*_	_*n*_*y*_*x*_	${\;}_{n}y_{x}^{s}$	_*n*_*N*_*x*_	_*n*_*Y*_*x*_	_*n*_*y*_*x*_	${\;}_{n}y_{x}^{s}$
< 1	8,490	848.6	0.0999	0.00138	8,165	915.6	0.1121	0.00155
1–4	31,922	301.2	0.0094	0.00052	30,767	90.8	0.0030	0.00016
5–14	62,511	204.7	0.0033	0.00048	59,534	206.9	0.0035	0.00051
15–24	66,947	2,300.1	0.0344	0.00476	64,744	618.3	0.0096	0.00132
25–34	175,515	4,722.9	0.0269	0.00365	168,896	1,566.4	0.0093	0.00126
35–44	169,625	9,420.0	0.0555	0.00903	134,598	3,084.4	0.0229	0.00373
45–54	119,555	15,185.3	0.1270	0.01713	105,231	6,946.9	0.0660	0.00890
55–64	76,742	13,781.8	0.1796	0.01567	76,907	7,556.9	0.0983	0.00857
65–74	49,239	11,170.6	0.2269	0.01498	56,924	8,363.8	0.1469	0.00970
75–84	33,375	11,171.3	0.3347	0.01501	47,799	12,643.9	0.2645	0.01186
85+	9,868	4,520.3	0.4581	0.00710	20,919	9,200.3	0.4398	0.00682

Total	803,789 (Pop.)	73,626.8 (*YLL*)	1.5557	0.08971 (*ASYR*)	774,484 (Pop.)	51,194.2 (*YLL*)	1.1758	0.05439 (*ASYR*)

**Table 6:** Expected years of life lost (YLL) and age-standardized YLL rates, By ethnicity, San Francisco, 2003–2004

Sex	Ethnicity	*YLL*	Deaths	*Average YLL*^*a*^	*ASYR*^*b*^	*ASYR *Ratio^c^
Male						
	African American	13,536.2	927	14.6	23,116.0	2.44
	American Indian	336.8	18	18.7	*	*
	Asian/Pacific Islander	14,846.4	1,594	9.3	5,589.0	0.59
	Latino/Hispanic	7,565.8	513	14.7	7,742.2	0.82
	White (reference)	36,442.8	3,201	11.4	9,459.0	1.00
	Multirace	705.8	46	15.3	5,031.1	0.53
	Other	56.2	4	14.0	*	*
	Missing	135.9	12	11.3	*	*
	
	Total	73,626.8	6,312		8971.1	
Female						
	African American	8,544.9	770	11.1	13,576.4	2.31
	American Indian	263.0	14	18.8	*	*
	Asian/Pacific Islander	13,363.0	1,509	8.9	3,915.1	0.67
	Latino/Hispanic	4,508.8	444	10.2	4,410.0	0.75
	White (reference)	24,080.0	2,963	8.1	5,867.0	1.00
	Multirace	353.0	25	14.1	2,719.8	0.46
	Other	16.7	1	16.7	*	*
	Missing	64.1	4	16.0	*	*
	
	Total	51,194.2	5,726		5438.6	

^*a *^Average *YLL *= *YLL *÷ Deaths

^*b *^*ASYR *= Age-standardized *YLL* rate per 100,000 persons per year

^*c *^Whites are reference groups for ratio comparison

* Rate was not calculated (less than 20 deaths or population estimate not available).

**Table 7:** Leading causes of premature death for San Francisco, By sex, 2003–2004

Rank	Underlying cause of death	*YLL*	*YLL *%^*a*^	Deaths	Average *YLL*^*b*^	*ASYR*^*c*^
Male						
1	Violence/assault, all mechanisms	2879.9	3.9	115	25.0	419.8
2	Drug overdose, unintentional	2908.1	3.9	134	21.7	301.8
3	HIV/AIDS	6464.6	8.8	319	20.3	673.1
4	Self-inflicted injuries, all mechanisms	3026.2	4.1	152	19.9	330.2
5	Alcohol use disorders	2228.5	3.0	128	17.4	245.8
6	Cirrhosis of the liver	1586.9	2.2	97	16.4	177.3
7	Liver cancer	2035.6	2.8	154	13.2	248.0
8	Hypertensive heart disease	3379.0	4.6	287	11.8	413.4
9	Diabetes mellitus	1656.5	2.2	147	11.3	198.8
10	Lung, bronchus, and trachea cancers	4134.3	5.6	387	10.7	515.8
11	Colon and rectum cancers	1394.7	1.9	136	10.3	173.5
12	Ischemic heart disease	9853.9	13.4	1103	8.9	1246.1
13	Chronic obstructive pulmonary disease	2241.5	3.0	269	8.3	293.1
14	Cerebrovascular disease	3420.2	4.6	418	8.2	439.3
15	Lower respiratory infections	1801.3	2.4	242	7.4	233.0
						
Female						
1	HIV/AIDS	823.6	1.6	36	22.9	101.7
2	Drug overdose, unintentional	843.8	1.6	37	22.8	96.6
3	Self-inflicted injuries, all mechanisms	992.9	1.9	48	20.7	123.9
4	Breast Cancer	2975.1	5.8	222	13.4	335.8
5	Pancreas cancer	1122.4	2.2	105	10.7	121.4
6	Lung, bronchus, and trachea cancers	3376.2	6.6	326	10.4	361.4
7	Lymphomas and multiple myeloma	852.0	1.7	86	9.9	89.8
8	Colon and rectum cancers	1407.7	2.7	153	9.2	145.3
9	Diabetes mellitus	1207.9	2.4	141	8.6	122.1
10	Hypertensive heart disease	2214.9	4.3	269	8.2	226.1
11	Chronic obstructive pulmonary disease	1651.8	3.2	211	7.8	166.0
12	Cerebrovascular disease	4221.3	8.2	614	6.9	406.6
13	Ischemic heart disease	6721.3	13.1	1017	6.6	646.3
14	Lower respiratory infections	1483.9	2.9	266	5.6	136.4
15	Alzheimer and other dementias	1414.1	2.8	305	4.6	118.3

^*a *^*YLL% *= *YLL *÷ Total *YLL *from all causes (Step 1: used to select top 15 causes)

^*b *^Average *YLL *= *YLL *÷ Deaths (Step 2: used to subrank top 15 causes)

^*c *^*ASYR *= Age-standardized *YLL* rate per 100,000 persons per year

### Cause of death categories

Using the International Classification of Diseases, 10th Revision (ICD-10) [14], the cause of death categories were adapted from the World Health Organization Global Burden of Disease Study [15] and the Centers for Disease Control and Prevention External Cause of Injury Mortality Matrix [16]. The cause of death category definitions were comprehensive, mutually exclusive, and sufficiently specific to support public health interventions [see Additional file 2]. For example, we used specific cancer diagnoses (e.g., "lung cancer") instead of broad categories (e.g., "all cancers").

### Interpolating standard model life table

For a single death at age *x*, the *YLL *for that individual is simply the expected years of life remaining at the age of death (i.e., life expectancy at age *x*: *e*_*x*_) based on the model life table West, Level 25 and 26 (Table 2). However, the table does not contain life expectancies for deaths within age intervals. For ages that fall within an interval, the life expectancy must be interpolated from the table.

For a group of deaths that occurred at ages within age interval *x *to *x *+ *n*, (i.e., *n *= age interval length), the expected years of life remaining for those deaths (${\;}_{n}e_{x}^{s}$) is estimated using a formula for linear interpolation (Equation 1) [17]:

(1) $${\;}_{n}e_{x}^{s}=e_{x}^{s}+{(}_{n}a_{x}-x)\frac{e_{x+n}^{s}-e_{x}^{s}}{(x+n)-x}$$

where _*n*_*a*_*x *_is the average age of death, and $e_{x}^{s}$ and $e_{x+n}^{s}$ are Table 2 model life expectancies at ages *x *and *x *+ *n*, respectively. See Table 4 for use of ${\;}_{n}e_{x}^{s}$ in spreadsheet calculations.

### Calculating expected years of life lost (*Y LL*)

For a group of deaths that occurred at ages within age interval *x *to *x *+ *n*, the crude expected years of life lost is

(2) $${\;}_{n}Y_{x}={(}_{n}D_{x}){(}_{n}e_{x}^{s})$$

where _*n*_*D*_*x *_is the number of deaths between age *x *and age *x *+ *n*.

To incorporate discounting and age weighting, one would use Equation 3:

(3) $${\;}_{n}Y_{x}={(}_{n}D_{x})[\frac{KCe^{r{(}_{n}a_{x})}}{{\left(r+\beta \right)}^{2}}\left(e^{z}\left[z-1\right]-e^{-{\left(r+\beta \right)}_{n}a_{x}}\left[-\left(r+\beta \right){\;}_{n}a_{x}-1\right]\right)+\frac{1-K}{r}\left(1-e^{r{(}_{n}e_{x}^{s})}\right)]$$

where $z=-(r+\beta ){(}_{n}e_{x}^{s}+{\;}_{n}a_{x})$. For this equation, *r *is the discount rate, and *β*, *C*, and *K *are age weighting constants (see Table 1 for complete definitions). To include age weighting, *K *(the modulation constant) can be set to 1. For this study, age weighting was not used (*K *= 0) and *r *= 0.03.

When the discount rate (*r*) is 0, Equation 3 simplifies to Equation 4:

(4) $${\;}_{n}Y_{x}={(}_{n}D_{x})[\frac{KCe^{-\beta {(}_{n}a_{x})}}{\beta^{2}}\left(e^{-\beta {(}_{n}e_{x}^{s})}\left[-\beta {(}_{n}e_{x}^{s}+{\;}_{n}a_{x})-1\right]-\left[-\beta {(}_{n}a_{x})-1\right]\right)+\left(1-K\right){(}_{n}e_{x}^{s})]$$

First, we calculated the expected of years of life lost, comparing men to women, by summing _*n*_*Y*_*x *_for all age intervals (Table 4):

(5) *YLL *= ∑_*n*_*Y*_*x*_

Using this approach, we calculated *Y LL*s for 117 specific causes of death stratified by sex, and stratified by sex and ethnicity.

### Calculating age-standardized expected years of life lost rates

Using the direct method [18], we calculated age standardized *YLL *rates (*ASYR*). First, we calculated age-specific rates of years of life lost (_*n*_*y*_*x*_). Then, these rates were reweighted using using the Year 2000 United States standard million population (_*n*_*w*_*x *_in Table 3) [18]. The reweighted rates (${\;}_{n}y_{x}^{s}$) were summed to get an ASYR (Equation 6).

(6) $$\begin{array}{c} ASYR=\sum {\;}_{n}y_{x}^{s}\\ =\sum {(}_{n}w_{x}){(}_{n}y_{x})\\ =\sum {(}_{n}w_{x})\left(\frac{{\;}_{n}Y_{x}}{{\;}_{n}N_{x}}\right) \end{array}$$

See Table 5 for use of Equation 6 in spreadsheet calculations.

### Ranking leading causes of premature death

Determining the leading causes of premature death required two steps. First, the leading 15 causes of death were ranked by *YLL *values for San Francisco (stratified by sex), and the leading 10 causes of death were ranked for each ethnic group (stratified by sex). Second, to highlight conditions of highly premature death, these were further subranked by their average *YLL *values. Age-standardized *YLL *rates were included to allow comparisons of ethnic groups within sex strata.

### Numerical computing

All analyses and graphics were conducted in R – a widely available, open source programming language for statistical computing and graphics [19]. To facilitate the *YLL *calculation for readers, we provide and demonstrate a numerical function for R [see Additional file 3].

## Results

Displayed in Table 4 is the spreadsheet format for calculating expected years of life lost (*YLL*) for San Francisco men and women. The age-interval specific number of deaths (_*n*_*D*_*x*_), average age of death (_*n*_*a*_*x*_), standard life expectancy (${\;}_{n}e_{x}^{s}$), and years of life lost (_*n*_*Y*_*x*_) are shown. In the years 2003–2004, 6312 men died with 73,627 years of life lost, and 5726 women died with 51,194 years of life lost.

**Figure 1:**
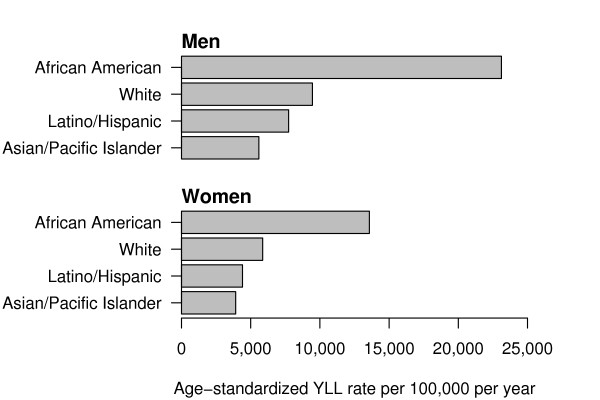
Comparison of age-standardized expected years of life lost rates (*ASYR*s), By sex and ethnicity, San Francisco, 2003–2004.

Displayed in Table 5 is the spreadsheet format for calculating direct age-standardized *YLL *rates (*ASYR*) for San Francisco men and women, combining years 2003–2004. The sex and age-interval specific population estimates (_*n*_*N*_*x*_), expected years of life lost (_*n*_*Y*_*x*_), expected *YLL *rate (_*n*_*y*_*x*_), and weighted expected *YLL *rate (${\;}_{n}y_{x}^{s}$) are displayed in each column. The *ASYR *for men was 65% higher compared to the *ASYR *for women (8971.1 per 100,000 persons per year vs. 5438.6 per 100,000 persons per year). Displayed in Table 6 are the *YLL*, number of deaths, average *YLL*, age-standardized *YLL *rates, and *ASYR *ratios stratified by sex and ethnicity. While whites and Asians account for the largest number of deaths (as expected based on population estimates), African American men and women have the highest age-standardized *YLL *rates (Figure 1). For all causes of death, the *ASYR *for African American men is 2.44 times higher compared to white men, and the *ASYR *for African American women is 2.31 times higher compared to white women.

The leading causes of premature death for San Francisco residents, ranked first by *YLL*s and then subranked by average *YLL*s, are displayed in Table 7. Among the top fifteen, the leading causes with the greatest degree of prematurity of premature deaths are those with the largest average *YLL*s. Therefore, among men, the leading causes of premature deaths were HIV/AIDS (average *YLL *= 20.3 years), suicide (19.9 years), drug overdose (21.7 years), homicide (25.0 years), and alcohol use disorder (17.4 years). The leading causes of deaths with the smallest average *YLL*s were ischemic heart disease (8.9 years), lung cancer (10.7 years), stroke (8.2 years), hypertensive heart disease (11.8 years), and chronic obstructive pulmonary disease (8.3 years).

Among women, the leading causes of premature deaths (those with the largest *YLL*s) were lung cancer (10.4 years), breast cancer (13.4 years), hypertensive heart disease (8.2 years), colon cancer (9.2 years), and diabetes mellitus (8.6 years). The leading causes of deaths with the smallest *YLL*s were ischemic heart disease (6.6 years), stroke (6.9 years), chronic obstructive pulmonary disease (7.8 years), pneumonia (5.6 years), and dementias (4.6 years).

Similarly, an analysis was conducted to rank the leading causes of premature death by ethnicity and sex [Additional file 1] for African Americans (Table A-1), Asians/Pacific Islanders (Table A-2), Latino/Hispanics (Table A-3), and whites (Table A-3). Similar analyses were done for each ethnic group. For example, among African American men, the leading causes of premature death (largest average *YLL*s) were homicide (25.9 years), HIV/AIDS (19.7 years), hypertensive heart disease (14.7 years), drug overdose (19.8 years), and alcohol use disorder (15.6 years). The leading causes of death with the smallest average *YLL*s were ischemic heart disease (11.8 years), lung cancer (13.1 years), stroke (10.7 years), chronic obstructive pulmonary disease (11.8 years), and diabetes mellitus (12.6 years).

**Figure 2:**
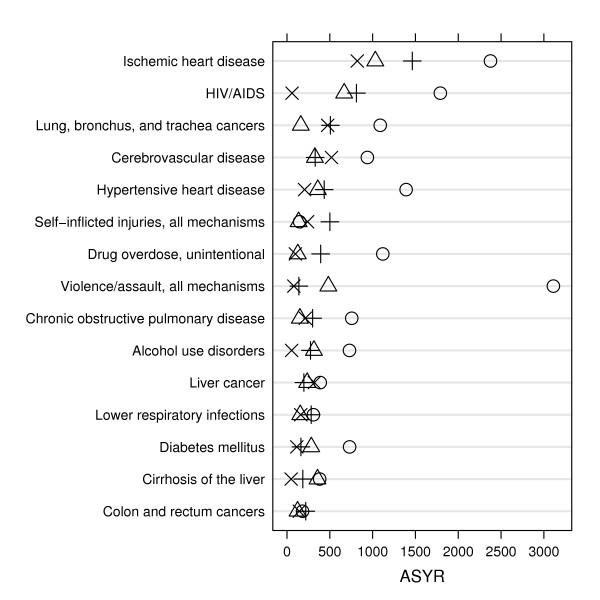
**Leading causes of premature death among men (ranked by *YLL*s), comparing age-standardized *YLL *rates (*ASYR*) by cause of death and ethnicity, San Francisco, 2003–2004.** Symbols: African American (○), Latino/Hispanic (△), Asian/Pacific Islander (×), White (+).

**Figure 3:**
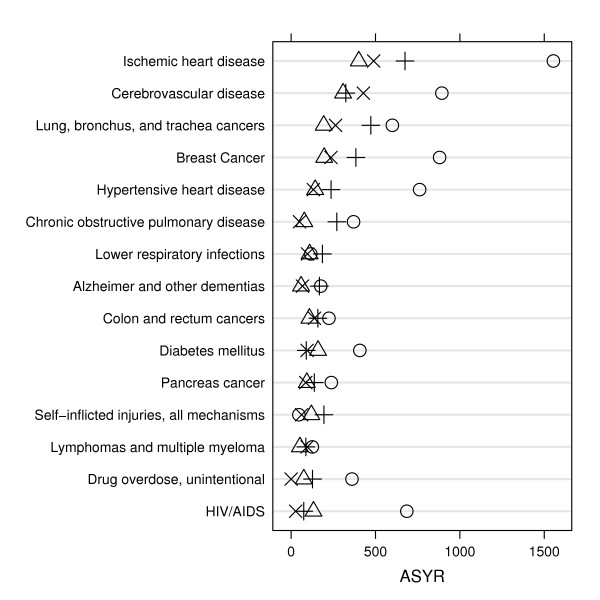
**Leading causes of premature death among women (ranked by *YLL*s), comparing age-standardized *YLL *rates (*ASYR*) by cause of death and ethnicity, San Francisco, 2003–2004.** Symbols: African American (○), Latino/Hispanic (△), Asian/Pacific Islander (×), White (+).

Age-standardized *YLL *rates (*ASYR*s) allow comparisons of the burden of premature mortality by ethnic group and specific cause of death (Figures 1, 2, 3). For example, for almost every leading cause of premature death in men and women, African Americans had the highest *ASYR*s compared to other ethnic groups. Among African American men, the disparity in *ASYR*s was most notable for violent assault (homicide), followed by HIV/AIDS, vascular diseases (ischemic and hypertensive heart, and cerebrovascular disease), accidental drug overdose, and lung cancer. Among African American women, the disparity in *ASYR*s was most notable for vascular diseases (ischemic and hypertensive heart, and cerebrovascular diseases), breast cancer, HIV/AIDS, and accidental drug overdose.

## Discussion

The key findings of this study are that (1) the leading causes of premature mortality were largely preventable: among men, these were HIV/AIDS, suicide, drug overdose, homicide, and alcohol use disorder; and among women, these were lung cancer, breast cancer, hypertensive heart disease, colon cancer, and diabetes mellitus; (2) leading causes of premature death differed remarkably between ethnic groups (Tables A-1–A-4); (3) a large health disparity was measured between African Americans and other ethnic groups: African American age-adjusted overall and cause-specific *YLL *rates are notably higher, especially for homicide among men (Figures 1, 2, and 3); and (4) except for homicide among Latino men, Latinos and Asians had comparable or lower *YLL *rates among the leading causes of premature death compared to whites (Figures 2 and 3). These results illustrate how death registry data can be used to measure, rank, and monitor the leading causes of premature mortality for a local geographic region. Such studies can be used to monitor the local mortality burden of disease and injury over time. For example, our results were compared to our previous San Francisco *YLL *study for the period 1990–1995 [11]. While the burden of HIV/AIDS deaths decreased remarkably, the ethnic health disparities remained, with African Americans continuing to suffer the largest burden. This was especially striking for homicides among African American men. The generally better health status of Asians and Latinos has persisted.

Several of these findings mirror those from national studies [20]. For example, the U.S. Burden of Disease and Injury Study [21] found many of the same preventable causes of premature death among the leading causes, and that the *YLL* ranking for each ethnic group was unique. Like our study, there were large disparities, measured as *DALY*s, between African Americans and other ethnic groups, and they reported better health outcomes among Asians than whites. The Eight Americas Study [22,23] also found large disparities, measured as life expectancy, between Asian Americans and African Americans. A recent examination of the U.S. black-white disparity in life expectancy during the period 1983–2003 [24] found, like our study, that cardiovascular disease (both males and females), homicide (males), and HIV/AIDS (males) were leading contributors to the gap in recent years.

Three measures were used in this study: *YLL*s, average *YLL*s, and *ASYR*s. The *YLL *is a stand-alone measure of mortality burden not requiring population estimates. It was used to rank the 15 leading causes of death for men and women (Table 7). However, these 15 leading causes were influenced by the larger number of deaths among older residents. To highlight premature, preventable causes of death, we then ranked these top 15 causes by their average *YLL*s. Notably, many of the leading causes of death have strong social determinants. Alternatively, the *ASYR *could have been used to rank the leading causes of death; however, this was not our first choice because it requires population estimates, and the rankings would still be influenced by older deaths. Given our availability of population estimates, *ASYR*s were used to make comparisons among ethnic groups (Table 6 and Additional file 1). However, only the *YLL*s (including average *YLL*s) were necessary to rank the leading causes of premature death. Similar analyses were conducted for each ethnic group [Additional file 1].

This study has several strengths. First, we used a simple measure of premature mortality – expected years of life lost – that can be calculated from death registry data that is readily available, population-based, and complete for the whole population. Second, *YLL *estimates can be calculated for a comprehensive list of causes of death. Third, *YLL *calculations do not require population estimates, allowing leading cause of deaths to be ranked for parts of the population (such as specific ethnicities or geographic areas) for which population estimates are not available. Fourth, subranking by average *YLL*s identifies leading causes of premature death, bringing attention to preventable deaths that contribute most to the mortality burden. Fifth, these analyses can be repeated periodically to monitor changes, guide and inform policy makers, and to direct and evaluate interventions.

Sixth, except for motor vehicle accidents [16], we used the Global Burden of Disease ICD-10 cause of death categories, making our methods similar to national and international studies [15,21]. Seventh, our study included Latinos/Hispanics, an important segment of the population that was not included in a similar national study [21]. Eighth, with the availability of ethnic-specific population estimates, we were able to age-standardize the *YLL*s to measure, compare, and monitor the ethnic health disparities in the burden of premature deaths. And ninth, our study findings are directly relevant and can be adapted to the diverse and unique needs of our communities, and to our local government and policymakers.

This study also has several limitations. First, the accuracy of data recorded on death certificates (e.g., underlying cause of death and ethnicity) varies by region and underlying cause [25]. Additionally, analyses using underlying cause of death categories may underestimate the mortality burden for selected contributing causes of death listed on the death certificates (e.g., diabetes mellitus) [26]. Second, the *YLL *metric does not measure well conditions that cause significant disease and disability, but are difficult to measure (e.g., mental illness) or do not result in death (e.g., osteoarthritis). Third, on average, there may be a 10-month or longer delay from the time a calendar year ends and the availability of ICD-10-coded death registry data.

Fourth, the ranking of a specific cause of death depends on its individual *YLL *magnitude as well as its relative contribution compared to other causes; changes in ranking for a cause over time may be due either to changes in the occurrence of that cause, or to changes in the occurrences of other causes ranked above or below it. Fifth, the average *YLL *could be large for a specific cause of death but only involve a small number of deaths (small burden). To avoid this problem, we only evaluated the average *YLL *for the highest ranked causes of death based on *YLL*s. Sixth, the *YLL *measure is not age-standardized and cannot be used to compare specific causes of death between groups with different age compositions. (With population estimates, *YLL *can be age-standardized as described in Methods.) And seventh, because of the uncertainty of population estimates, age-standardized rates must also be interpreted with caution. In spite of these limitations, using *YLL*s to rank the leading causes of premature death provides community residents, community-based organizations, policy makers, public health authorities, and researchers with local, representative, objective, and informative data to guide and inform public health priorities, and to direct and evaluate public health interventions.

This study has the following key implications: First, we provide the methodological details for calculating *YLL *to measure the burden of premature mortality for any geographic area that has death registry data. We provide both the ICD-10 causes of death classifications used for this study [Additional file 2] and the computational program code for calculating age-interval-specific expected years of life lost that can incorporate discounting (used in this study) and age weighting (not used in this study) [Additional file 3]. This code can be executed in a freely available, open source program for statistical computing and graphics [19]. And second, we demonstrate how these results can be used to rank the leading cause of premature death for major ethnic groups. The rankings can be use to guide, inform, and monitor public health priorities and programs for each group. These analyses can become part of routine public health surveillance for local health jurisdictions, as we have done in San Francisco.

## Conclusion

Population health measures based on *YLL*s are readily calculated and useful for measuring, ranking, and monitoring the leading causes of premature death for a local geographic area, and for measuring and monitoring the impact of local efforts to reduce premature mortality in ethnic groups for which there are health disparities.

## Competing interests

The author(s) declare that they have no competing interests.

## Authors' contributions

TJA conceived and designed the study, conducted the analyses, and prepared the initial manuscript. DYL reviewed the literature on Global Burden of Disease Study methods and applied the findings to our study. BSK reviewed the literature on Global Burden of Disease Study methods and local area research studies, and applied findings to our cause of death classifications. RR assisted in statistical programming, quality control, review of quantitative methods, and development of population health applicability of these measures. MHK reviewed the study for clinical accuracy, epidemiologic methods, and public health impact. All authors contributed substantially to the interpretation of findings and manuscript revisions. All authors read and approved the final manuscript.

## Pre-publication history

The pre-publication history for this paper can be accessed here:



## Supplementary Material

Additional file 1 **Leading causes of premature death by ethnicity and sex, San Francisco, 2003–2004**. This file contains tables for the leading causes of premature death by ethnicity and sex. We illustrate how these methods can be repeated for population subgroups to inform and guide public health priorities. Click here for file

Additional file 2 **Cause of death classification using International Classification of Diseases, 10th Revision (ICD-10) codes**. This file contains the ICD-10 codes used for our cause of death categories, which were adapted from the World Health Organization Global Burden of Disease Study [15] and the Centers for Disease Control and Prevention External Cause of Injury Mortality Matrix [16]. Click here for file

Additional file 3 **Open source programming code for calculating expected years of life lost**. This file contains R programming code to calculate expected years of life lost. R is a comprehensive, open source software package for statistical computing and graphics [19]. Click here for file
