# Supplementary material for: Calculating expected years of life lost for assessing local ethnic disparities in causes of premature death
Source: BMC Public Health. 2008 Apr 10;8:116. doi: 10.1186/1471-2458-8-116 (PMC2386472; doi:10.1186/1471-2458-8-116)
Supplement: Additional file 1 — Leading causes of premature death by ethnicity and sex, San Francisco, 2003–2004. This file contains tables for the leading causes of premature death by ethnicity and sex. We illustrate how these methods can be repeated for population subgroups to inform and guide public health priorities. [file 1471-2458-8-116-S1.pdf]

Additional file 1 — Leading causes of premature death by ethnicity and sex, San Francisco, 2003-2004

Tomás J. Aragón, MD, DrPH

December 28, 2007

Table A-1: Leading causes of premature death for African Americans, San Francisco, 2003–2004

Rank	Underlying cause of death	YLL	YLL %[a]	Deaths	Average YLL[b]	$ASYR$[c,d]
Male						
1	Violence/assault, all mechanisms	1607.8	11.9	62	25.9	3110.9
2	Drug overdose, unintentional	711.5	5.3	36	19.8	1118.8
3	HIV/AIDS	1183.7	8.7	60	19.7	1790.7
4	Alcohol use disorders	468.4	3.5	30	15.6	729.7
5	Hypertensive heart disease	882.2	6.5	60	14.7	1391.5
6	Lung, bronchus, and trachea cancers	679.7	5.0	52	13.1	1088.8
7	Diabetes mellitus	429.5	3.2	34	12.6	730.9
8	Ischemic heart disease	1420.5	10.5	120	11.8	2376.2
9	Chronic obstructive pulmonary disease	460.1	3.4	39	11.8	755.9
10	Cerebrovascular disease	557.2	4.1	52	10.7	938.7
Female						
1	Drug overdose, unintentional	203.4	2.4	9	22.6	360.5
2	HIV/AIDS	379.9	4.4	17	22.3	685.6
3	Breast Cancer	554.8	6.5	39	14.2	880.0
4	Nephritis and nephrosis	217.0	2.5	18	12.1	338.1
5	Lung, bronchus, and trachea cancers	422.0	4.9	36	11.7	599.9
6	Chronic obstructive pulmonary disease	244.7	2.9	21	11.7	369.9
7	Hypertensive heart disease	525.9	6.2	49	10.7	761.5
8	Diabetes mellitus	296.3	3.5	32	9.3	407.0
9	Ischemic heart disease	1161.4	13.6	137	8.5	1554.4
10	Cerebrovascular disease	674.4	7.9	82	8.2	893.2

[a] $YLL\% = YLL \div$ Total YLL from all causes (Step 1: used to select top 10 causes)
[b] Average $YLL = YLL \div$ Deaths (Step 2: used to subrank top 10 causes)
[c] $ASYR =$ Age-standardized YLL rate per 100,000 persons per year
[d] Rates calculated from less than 20 deaths may be unreliable.

Table A-2: Leading causes of premature death for Asians/Pacific Islanders, San Francisco, 2003–2004

Rank	Underlying cause of death	YLL	YLL %[a]	Deaths	Average YLL[b]	$ASYR$[c,d]
Male						
1	Self-inflicted injuries, all mechanisms	613.8	4.1	31	19.8	240.0
2	Liver cancer	883.2	5.9	71	12.4	320.0
3	Lymphomas and multiple myeloma	334.9	2.3	33	10.1	121.6
4	Hypertensive heart disease	564.7	3.8	58	9.7	205.3
5	Colon and rectum cancers	395.5	2.7	41	9.6	143.8
6	Lung, bronchus, and trachea cancers	1345.6	9.1	144	9.3	475.3
7	Cerebrovascular disease	1409.7	9.5	168	8.4	519.1
8	Ischemic heart disease	2204.6	14.8	289	7.6	820.9
9	Chronic obstructive pulmonary disease	598.3	4.0	88	6.8	213.5
10	Lower respiratory infections	434.9	2.9	73	6.0	160.3
Female						
1	Breast Cancer	752.6	5.6	49	15.4	235.9
2	Liver cancer	372.7	2.8	34	11.0	103.5
3	Lymphomas and multiple myeloma	346.7	2.6	33	10.5	95.0
4	Lung, bronchus, and trachea cancers	957.8	7.2	93	10.3	263.4
5	Colon and rectum cancers	491.8	3.7	48	10.2	138.1
6	Hypertensive heart disease	514.1	3.8	67	7.7	131.4
7	Diabetes mellitus	363.0	2.7	49	7.4	94.5
8	Cerebrovascular disease	1623.6	12.2	221	7.3	428.1
9	Ischemic heart disease	1860.7	13.9	265	7.0	489.0
10	Lower respiratory infections	378.1	2.8	69	5.5	96.9

[a] $YLL\% = YLL \div$ Total YLL from all causes (Step 1: used to select top 10 causes)
[b] Average $YLL = YLL \div$ Deaths (Step 2: used to subrank top 10 causes)
[c] $ASYR =$ Age-standardized YLL rate per 100,000 persons per year
[d] Rates calculated from less than 20 deaths may be unreliable.

Table A-3: Leading causes of premature death for Latinos/Hispanics, San Francisco, 2003–2004

Rank	Underlying cause of death	YLL	YLL %[a]	Deaths	Average YLL[b]	$ASYR$[c,d]
Male						
1	Violence/assault, all mechanisms	582.8	7.7	22	26.5	482.7
2	Self-inflicted injuries, all mechanisms	213.1	2.8	9	23.7	135.2
3	HIV/AIDS	888.4	11.7	41	21.7	667.7
4	Alcohol use disorders	384.7	5.1	19	20.2	314.5
5	Cirrhosis of the liver	354.5	4.7	20	17.7	356.2
6	Liver cancer	203.7	2.7	14	14.5	235.5
7	Diabetes mellitus	257.3	3.4	19	13.5	283.3
8	Hypertensive heart disease	283.9	3.8	22	12.9	359.8
9	Ischemic heart disease	813.4	10.8	78	10.4	1031.4
10	Cerebrovascular disease	249.8	3.3	25	10.0	325.6
Female						
1	Self-inflicted injuries, all mechanisms	124.6	2.8	5	24.9	119.8
2	HIV/AIDS	138.5	3.1	6	23.1	132.4
3	Breast Cancer	202.2	4.5	14	14.4	195.8
4	Cirrhosis of the liver	226.0	5.0	18	12.6	230.7
5	Lung, bronchus, and trachea cancers	197.0	4.4	19	10.4	193.1
6	Diabetes mellitus	161.6	3.6	17	9.5	159.4
7	Hypertensive heart disease	144.7	3.2	19	7.6	142.3
8	Ischemic heart disease	418.9	9.3	60	7.0	401.0
9	Cerebrovascular disease	322.2	7.1	46	7.0	307.8
10	Lower respiratory infections	113.1	2.5	19	6.0	109.4

[a] $YLL\% = YLL \div$ Total YLL from all causes (Step 1: used to select top 10 causes)
[b] Average $YLL = YLL \div$ Deaths (Step 2: used to subrank top 10 causes)
[c] $ASYR$ = Age-standardized YLL rate per 100,000 persons per year
[d] Rates calculated from less than 20 deaths may be unreliable.

Table A-4: Leading causes of premature death for Whites, San Francisco, 2003–2004

Rank	Underlying cause of death	YLL	YLL %[a]	Deaths	Average YLL[b]	$ASYR$[c,d]
Male						
1	Drug overdose, unintentional	1670.9	4.6	74	22.6	393.4
2	HIV/AIDS	3975.1	10.9	198	20.1	810.9
3	Self-inflicted injuries, all mechanisms	2006.4	5.5	102	19.7	500.8
4	Alcohol use disorders	1196.2	3.3	69	17.3	274.2
5	Hypertensive heart disease	1582.9	4.3	143	11.1	434.0
6	Lung, bronchus, and trachea cancers	1913.9	5.3	175	10.9	506.5
7	Ischemic heart disease	5339.0	14.7	607	8.8	1463.4
8	Chronic obstructive pulmonary disease	1041.2	2.9	126	8.3	299.0
9	Lower respiratory infections	1039.2	2.9	137	7.6	282.5
10	Cerebrovascular disease	1156.3	3.2	168	6.9	329.2
Female						
1	Self-inflicted injuries, all mechanisms	676.9	2.8	32	21.2	194.3
2	Breast Cancer	1416.1	5.9	118	12.0	383.8
3	Lung, bronchus, and trachea cancers	1780.1	7.4	177	10.1	472.2
4	Colon and rectum cancers	647.6	2.7	80	8.1	158.5
5	Hypertensive heart disease	994.0	4.1	131	7.6	236.4
6	Chronic obstructive pulmonary disease	1139.6	4.7	152	7.5	270.8
7	Cerebrovascular disease	1556.2	6.5	262	5.9	324.0
8	Ischemic heart disease	3179.5	13.2	547	5.8	674.9
9	Lower respiratory infections	887.7	3.7	159	5.6	185.4
10	Alzheimer and other dementias	914.7	3.8	193	4.7	167.7

[a] $YLL\% = YLL \div$ Total YLL from all causes (Step 1: used to select top 10 causes)
[b] Average $YLL = YLL \div$ Deaths (Step 2: used to subrank top 10 causes)
[c] $ASYR =$ Age-standardized YLL rate per 100,000 persons per year
[d] Rates calculated from less than 20 deaths may be unreliable.
