# Supplementary material for: Calculating expected years of life lost for assessing local ethnic disparities in causes of premature death
Source: BMC Public Health. 2008 Apr 10;8:116. doi: 10.1186/1471-2458-8-116 (PMC2386472; doi:10.1186/1471-2458-8-116)
Supplement: Additional file 2 — Cause of death classification using International Classification of Diseases, 10th Revision (ICD-10) codes. This file contains the ICD-10 codes used for our cause of death categories, which were adapted from the World Health Organization Global Burden of Disease Study [15] and the Centers for Disease Control and Prevention External Cause of Injury Mortality Matrix [16]. [file 1471-2458-8-116-S2.pdf]

Additional file 2 — Cause of death classification using International Classification of Diseases, 10th Revision (ICD-10) codes

Tomás J. Aragón, MD, DrPH

December 28, 2007

Our cause of death categories were based on the Global Burden of Disease Study ICD-10 classification scheme.

Code	Underlying cause of death	ICD-10 codes
U003	Tuberculosis	A15–A19, B90
U005	Syphilis	A50–A53
U006	Chlamydia	A55–A56
U007	Gonorrhea	A54
U008	Other STDs	A57–A64, N70–N73
U009	HIV/AIDS	B20–B24
U010	Diarrheal Diseases	A00, A01, A03, A04, A06–A09
U012	Pertussis	A37
U013	Poliomyelitis	A80, B91
U014	Diphtheria	A36
U015	Measles	B05
U016	Tetanus	A33–A35
U017	Meningitis	A39, G00, G03
U017.5	Hepatitis A	B15
U018	Hepatitis B	B16–B19 (minus B17.1, B18.2)
U019	Hepatitis C	B17.1, B18.2
U020	Malaria	B50–B54
U021	Tropical-cluster diseases	B55–B57, B65, B73, B74.0–B74.2
U028	Leprosy	A30
U029	Dengue	A90–A91
U030	Viral encephalitis, mosquito-borne	A83
U031	Trachoma	A71
U032	Intestinal nematode infections	B76–B81
U039	Lower respiratory infections	J10–J18, J20–J22
U040	Upper respiratory infections	J00–J06
U041	Otitis media	H65–H66
U043	Maternal hemorrhage	O44–O46, O67, O72
U044	Maternal sepsis	O85–O86
U045	Hypertensive disorders of pregnancy	O10–O16
U046	Obstructed labor	O64–O66
U047	Abortion	O00–O07

Table 1: (continued)

Code	Underlying cause of death	ICD-10 code
U048	Other maternal conditions	O20–O43, O47–O63, O68–O71, O73–O75, O87–O99
U050	Low birth weight	P05–P07
U051	Birth asphyxia and birth trauma	P03, P10–P15, P20–P29
U052	Other perinatal conditions	P00–P02, P04, P08, P35–P96
U053	Nutritional disorders	E00–E02, E40–E46, E50, D50–D53, D64.9, E51–E64
U061	Mouth and oropharynx cancers	C00-C14
U062	Esophagus cancer	C15
U063	Stomach cancer	C16
U064	Colon and rectum cancers	C18–C21
U065	Liver cancer	C22
U066	Pancreas cancer	C25
U067	Lung, bronchus, and trachea cancers	C33–C34
U068	Melanoma and other skin cancers	C43–C44
U069	Breast Cancer	C50
U070	Cervix uteri cancer	C53
U071	Corpus uteri cancer	C54–C55
U072	Ovary cancer	C56
U073	Prostate cancer	C61
U074	Bladder cancer	C67
U075	Lymphomas and multiple myeloma	C81–C90, C96
U076	Leukemia	C91–C95
U077	Other malignant neoplasms	C17, C23, C24, C26–C32, C37–C41, C45–C49, C51, C52, C57–C60, C62–C66, C68–C80, C97
U078	Other neoplasms, non-malignant	D00–D48
U079	Diabetes mellitus	E10–E14
U080	Endocrine, metabolic, immune disorders	D55–D64 (minus D64.9), D65–D89, E03–E07, E15–E16, E20–E34, E65–E88
U082	Unipolar depressive disorders	F32–F33
U083	Bipolar affective disorder	F30–F31
U084	Schizophrenia	F20–F29
U085	Epilepsy	G40–G41
U086	Alcohol use disorders	F10
U087	Alzheimer and other dementias	F01, F03, G30–G31
U088	Parkinson disease	G20–G21
U089	Multiple sclerosis	G35
U090	Drug use disorders	F11–F16, F18–F19
U091	Post-traumatic stress disorder	F43.1
U092	Obsessive-compulsive disorder	F42
U093	Panic disorder	F40.0, F41.0
U094	Insomnia (primary)	F51
U095	Migraine	G43
U096	Mental Retardation due to lead exposure	F70–F79
U097	Other neuropsychiatric disorders	F04–F09, F17, F34–F39,

Table 1: (continued)

Code	Underlying cause of death	ICD-10 code
		F401–F409, F411–F419, F43 (minus F43.1), F44–F50, F52–F69, F80–F99, G06–G12, G23–G25, G36, G37, G44–G98
U099	Glaucoma	H40
U100	Cataracts	H25–H26
U103	Other sense organs disorders	H00–H21, H27–H35, H43–H61 (minus H524), H68–H83, H92–0H93
U105	Rheumatic heart disease	I01–I09
U106	Hypertensive heart disease	I10–I13
U107	Ischemic heart disease	I20–I25
U108	Cerebrovascular disease	I60–I69
U109	Inflammatory heart disease	I30–I33, I38, I40, I42
U110	Other cardiovascular diseases	I00, I26–I28, I34–I37, I44–I51, I70–I99
U112	Chronic obstructive pulmonary disease	J40–J44
U113	Asthma	J45–J46
U114	Other respiratory diseases	J30–J39, J47–J98
U116	Peptic ulcer disease	K25–K27
U117	Cirrhosis of the liver	K70, K74
U118	Appendicitis	K35–K37
U119	Other digestive diseases	K20–K22, K28–K31, K38, K40–K66, K71–K73, K75–K92
U121	Nephritis and nephrosis	N00–N19
U122	Benign prostatic hypertrophy	N40
U123	Other genitourinary system diseases	N20–N39, N41–N64, N75–N98
U124	Skin diseases	L00–L98
U126	Rheumatoid arthritis	M05–M06
U127	Osteoarthritis	M15–M19
U128	Gout	M10
U129	Low back pain	M45–M48, M54 (minus M54.2)
U130	Other musculoskeletal disorders	M00–M02, M08, M11–M13, M20–M43, M50–M53, M54.2, M55–M99
U131	Congenital anomalies	Q00–Q99
U143	Oral conditions	K00-K14
U150.1	Motor vehicle accident-Occupant	V30–V39 (.4–.9), V40–V49 (.4–.9), V50–V59 (.4–.9), V60–V69 (.4–.9), V70–V79 (.4–.9), V83–V86 (.0–.3)
U150.2	Motor vehicle accident-Motorcyclist	V20–V28 (.3–.9), V29 (.4–.9)
U150.3	Motor vehicle accident-Pedal cyclist	V12–V14 (.3–.9), V19 (.4–.6)
U150.4	Motor vehicle accident-Pedestrian	V02–V04 (.1, .9), V09.2
U150.5	Motor vehicle accident-Other	V80 (.3–.5), V81.1, V82.1

Table 1: (continued)

Code	Underlying cause of death	ICD-10 code
U150.6	Motor vehicle accident-Unspecified	V87 (.0–.8), V89.2
U150.7	Pedal cyclist, other	V10–V11, V12–V14 (.0–.2), V15–V18, V19 (.0–.3, .8, .9)
U150.8	Pedestrian, other	V01, V02–V04 (.0), V05, V06, V09 (.0, .1, .3, .9)
U151	Drug overdose, unintentional	X40–X49
U152	Falls, unintentional	W00–W19
U153	Fires, unintentional	X00–X09
U154	Drownings, unintentional/unknown	W65–W74, Y21
U155.1	Medical care and drug adverse effects	Y40–Y59, Y60–Y84, Y88
U157	Self-inflicted injuries, all mechanisms	X60–X84, Y870
U158	Violence/assault, all mechanisms	X85–Y09, Y871
U159	War operations	Y36
U160	Legal intervention, all mechanisms	Y35
U999	Other causes (not categorized)	ICD-10 codes not above
