# Supplementary material for: Calculating expected years of life lost for assessing local ethnic disparities in causes of premature death
Source: BMC Public Health. 2008 Apr 10;8:116. doi: 10.1186/1471-2458-8-116 (PMC2386472; doi:10.1186/1471-2458-8-116)
Supplement: Additional file 3 — Open source programming code for calculating expected years of life lost. This file contains R programming code to calculate expected years of life lost. R is a comprehensive, open source software package for statistical computing and graphics [19]. [file 1471-2458-8-116-S3.pdf]

Additional file 3: Open source programming code for calculating expected years of life lost

Tomás J. Aragón, MD, DrPH

December 28, 2007

R is a comprehensive, open source software package for statistical computing and graphics.[1] R was developed and is maintained by university-affiliated statistical programers from around the world. Corresponding to Equations 3 and 4, below is the R function used to calculate the YLLs for this study:

```
yll <- function(number.deaths, average.age.death, model.life.expectancy,
                discount.rate = 0.03, beta.constant = 0.04,
                modulation.constant = 0, adjustment.constant = 0.1658){
  ##abbreviate inputs
  N <- number.deaths;          a <- average.age.death
  L <- model.life.expectancy; r <- discount.rate
  b <- beta.constant;         K <- modulation.constant
  CC <- adjustment.constant
  ##do calculations
  if(discount.rate==0){
    N*(K*CC*((exp(-b*a))/b^2)*((exp(-b*L))*
                              (-b*(L+a)-1)-(-b*a-1))+((1-K)*L))
  } else {
    N*(K*((CC*exp(r*a))/(-(r+b)^2))*((exp(-(r+b)*(L+a))*(-(r+b)*
      (L+a)-1))-(exp(-(r+b)*a)*(-(r+b)*a-1)))+((1-K)/r)*((1-exp(-r*L))))
  }
}
```

For age interval x to $x+n$: to calculate the expected years of life lost for this age interval (${}_nY_x$), we only need the number of deaths in that interval (${}_nD_x$), the average age of death for deaths in that interval (${}_na_x$), and the corresponding interpolated model life expectancy for that interval (${}_ne^s_x$) using Equation 1. To use this function, we must provide these input values. The other parameters have their default values; for example, the discounting rate parameter is set to 0.03 (3%).

For example, for the years 2003–2004 in San Francisco, for men ages 50 to 54 years (see Table 4), there were 434 deaths with an average age of death of 52.5 years. The interpolated model life expectancy for that age interval was 28.6 years. Once the function has been loaded into, we only need to provide the first three values. Here is the calculation in R, including changing the discounting rate:

```
> yll(434, 52.5, 28.6)
[1] 8332.666
> yll(434, 52.5, 28.6, discount.rate=0)
[1] 12412.4
```

The expected years of life lost for San Francisco male deaths, ages 50 to 54 years, during years 2003–2004, was 8,332.7 years with discounting and 12,412.4 years without discounting. The value 8,332.7 differs from our study results due to rounding error.

[1] http://www.r-project.org
